# EEG Identity Authentication in Multi-Domain Features: A Multi-Scale 3D-CNN Approach

**DOI:** 10.3389/fnbot.2022.901765

**Published:** 2022-06-16

**Authors:** Rongkai Zhang, Ying Zeng, Li Tong, Jun Shu, Runnan Lu, Zhongrui Li, Kai Yang, Bin Yan

**Affiliations:** ^1^Henan Key Laboratory of Imaging and Intelligent Processing, People's Liberation Army (PLA) Strategic Support Force Information Engineering University, Zhengzhou, China; ^2^Key Laboratory for Neuro Information of Ministry of Education, School of Life Science and Technology, University of Electronic Science and Technology of China, Chengdu, China

**Keywords:** EEG, identity authentication, multi-scale, 3D-CNN, ERP

## Abstract

Electroencephalogram (EEG) authentication has become a research hotspot in the field of information security due to its advantages of living, internal, and anti-stress. However, the performance of identity authentication system is limited by the inherent attributes of EEG, such as low SNR, low stability, and strong randomness. Researchers generally believe that the in-depth fusion of features can improve the performance of identity authentication and have explored among various feature domains. This experiment invited 70 subjects to participate in the EEG identity authentication task, and the experimental materials were visual stimuli of the self and non-self-names. This paper proposes an innovative EEG authentication framework, including efficient three-dimensional representation of EEG signals, multi-scale convolution structure, and the combination of multiple authentication strategies. In this work, individual EEG signals are converted into spatial–temporal–frequency domain three-dimensional forms to provide multi-angle mixed feature representation. Then, the individual identity features are extracted by the various convolution kernel of multi-scale vision, and the strategy of combining multiple convolution kernels is explored. The results show that the small-size and long-shape convolution kernel is suitable for ERP tasks, which can obtain better convergence and accuracy. The experimental results show that the classification performance of the proposed framework is excellent, and the multi-scale convolution method is effective to extract high-quality identity characteristics across feature domains. The results show that the branch number matches the EEG component number can obtain the excellent cost performance. In addition, this paper explores the network training performance for multi-scale module combination strategy and provides reference for deep network construction strategy of EEG signal processing.

## Introduction

Electroencephalogram (EEG) identity authentication extracts the neural activity pattern of the user's brain and applies the EEG signal as a biological feature to the individual identity recognition system. Modern brain science research shows (Nakamura et al., 2017) that people brains have not only the structural differences determined by genes such as fingerprints and faces, but also functional differences in memory, personality, and thinking patterns. EEG has unique advantages in the field of biometric identification, such as *in vivo*, stress resistance, and internality. As a high-level security authentication method, EEG has become a research hotspot. A large number of studies (Marcel and Millan, 2007; Alariki et al., 2018) have verified the possibility of EEG identifying individuals. Individuals show significant individual differences under both evoked (Rathi et al., 2020) and spontaneous (Thomas and Vinod, 2017) tasks.

However, the randomness and weak signal-to-noise ratio of EEG signals restrained the performance of identity authentication, and the current mainstream feature extraction methods have limited improvement in classification performance. It is an urgent problem to improve the performance of EEG identity authentication by constructing fusion features to comprehensively characterize individual EEG features.

Researchers explored a variety of feature fusion methods. In the field of traditional machine learning (Palaniappan and Mandic, 2007), the sensor domain and spatial domain features were spliced, and the fused EEG features effectively improved the classification accuracy. In addition (Arvind et al., 2012), multiple feature domains were arranged and combined. Linear combination (Zhang et al., 2021) was applied in the feature space. Yuan et al. (2018) focused on the time series information of EEG to extract the features through the contextual semantics of EEG, and the proposed method improved the performance of epileptic seizure detection. The above studies have improved the performance of EEG data features.

In this paper, an innovative 3D input representation and multi-scale vision CNN framework is proposed for EEG identity authentication task, which effectively integrates spatial, temporal, and frequency domains in EEG features. The 3D representation of EEG signals comprehensively analyzes the individual's brain identity features from multiple perspectives, and the hybrid mode across feature domains is more integrated and diverse. The multi-scale convolution kernel extracts the brain identity information from different views and characterizes the individual identity by analyzing the individual characteristics of EEG signals from different fields of vision. Furthermore, we discuss the performance and combining strategies of multiple convolution kernels. The proposed multi-scale CNN framework of mixed features significantly improves the performance of EEG authentication. On the feature level, the mixed features integrate the multi-angle information of the research object. On the network level, multi-scale networks further expand the advantages of parallel feature extraction. The proposed method has broad application prospects in dynamic timing information processing, such as video data processing, satellite image analysis, gait recognition, and so on.

The main contributions of this paper are as follows:

(1) An efficient 3D EEG data fusion representation method is proposed to characterize EEG features from multiple perspectives in the spatial, temporal, and frequency domains.
(2) The convolution kernel scale and combination strategy suitable for EEG data were explored, and the rectangular convolution kernel that prefers temporal features has better EEG extraction performance.
(3) The depth of multi-scale modules and the branch number are studied. The branch number should match the number of EEG components, and the shallow multi-branch structure has high application cost performance.

## Background

There were some common methods of individual identity in spatial, time, and frequency domains. For example, event-related potential (ERP) components were extracted in time domain (Zeng et al., 2018), power spectral density (PSD) energy of each frequency band in frequency domain (Harshit et al., 2016), and common spatial pattern (CSP) method in spatial domain (Jayarathne et al., 2016). The research results showed that the classification performance of mixed feature domain concatenation was better than single feature domain, and feature fusion methods relying on researchers' experience have been rapidly developed. The above research provided a valuable reference, but the fusion degree of feature domain and feature diversity is still needed to be improved.

In addition to relying on empirical combination features, with the gradual rise of end-to-end learning mode of deep learning framework, automatic and non-linear feature fusion methods based on neural network were widely used, and the efficiency and performance of fusion features were better than traditional empirical methods. Specifically, after the application of multi-scale convolution model in two-dimensional image processing, the features of different scale fields were automatically extracted and fused by parallel structure. We summarized that the latest research improves the input form to three-dimensional, and the convolution neural network (CNN) performed rich, comprehensive, and complementary feature domain fusion. The multi-scale convolution network in the field of EEG had a lot of exploration. The application scenarios included emotion recognition, fatigue driving (Cho and Hwang, 2020), epilepsy prediction (Ozcan and Erturk, 2019), and motor imagery (Zhang et al., 2019) tasks.

### EEG Presentation Form

For 3D CNN, several methods were designed to convert scalp EEG signals into 3D presentation forms. Common dimensions were assigned to the main features of two dimensions and the auxiliary features of one dimension. Zhao et al. (2019a) demonstrated that in the motor imagery task, the strategy of electrode combination was studied. According to the electrode position, it was mapped to a 3 × 3 or 4 × 4 two-dimensional plane to form spatial features, and another dimension shows temporal features. In Deng et al. (2021) research, a two-dimensional 9 × 9 spatial representation matrix was designed for 32-channel electrodes, and the other dimension was the temporal domain signal of EEG array. Zhang et al. (2019) combined 2D temporal features and 1D frequency feature in the motor imagery task to form a three-dimensional feature domain, representing the time-frequency feature tensor of EEG. In the study of psychological load (Kwak et al., 2020), EEG signals were presented as 2D spatial information and 1D frequency information and focused on the rhythm characteristics of EEG frequency band.

For different cognitive tasks, 3D feature combination focuses on two kinds of temporal, frequency, and spatial domains and abandons a feature domain as a cost, which is not fully utilized. The rich temporal, frequency, and spatial features of EEG data have not been fully excavated, and more efficient 3D feature domain fusion method remains to be explored.

### Multi-Scale Convolution Module

The three-dimensional convolution model applied in the field of EEG is still dominated by single convolution kernel and serial stack architecture. Inadequate advanced studies have found that the performance of parallel multi-scale convolution structure was better than serial mode, and the parallel structure of multi-scale is beneficial to capture the characteristics of different scales. The inception module (Lee et al., 2020) in the field of image was used in the task of brain control manipulator to obtain better stability and accuracy in decoding motion intention. In addition, Zhao et al. (2019b) research designed small receptive field network (SRF), medium receptive field network (MRF), and large receptive field network (LRF), using multi-scale three-dimensional dimensions (2 × 2 × 1, 2 × 2 × 3, 2 × 2 × 5) to extract EEG features.

The combination strategy of convolution kernel continues the experience in the field of image, without improving the characteristics of EEG. Moreover, insufficient study researched on the performance of different convolution sizes in the EEG field, and the editing and parameter setting were based on the researcher experience. The above research shows that the fusion feature domain of EEG identity authentication is not sufficient and diverse, and the performance of single convolution kernel and multi-scale combination strategy needs to be studied.

## Methods

### Database

**Figure 1:**
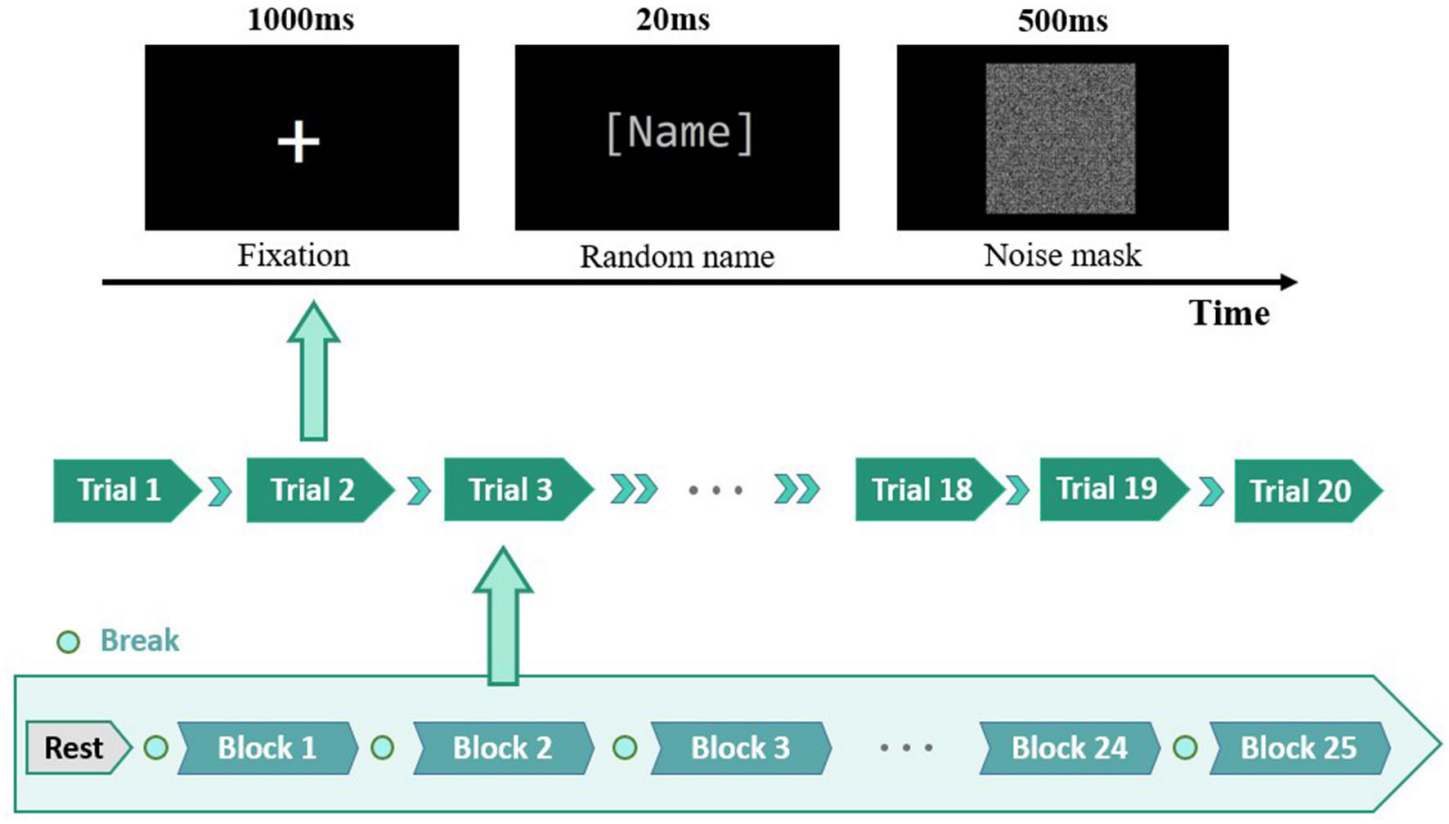
Task paradigm of EEG authentication.

As shown in Figure 1, the EEG authentication task (Zhang et al., 2022) was to distinguish between the self and non-self-names. The oddball paradigm randomly showed 500 names, including 100 subjects' own names, 150 familiar names, 300 strangers' names, and 50 blank names. The acquaintances were the names of the subjects' family members or closest friends, which were provided by the subjects before the experiment. The names of acquaintances interfere greatly with the subjects, which was used as a supplement to the control of non-self-names in the experiment. Strange names were randomly selected from the citizen name database. The subjects confirmed that each stranger was unfamiliar before the experiment. Blank name was black background and no text. To eliminate the influence of cross and mask factors on participants, the study only retained the brain response of name stimuli. In the preprocessing stage, the blank name was subtracted as the name response baseline of the brain. First, the blank names of all subjects were collected and averaged globally. Second, the global average of blank names was subtracted from the single trial of each self, familiar, and stranger name.

**Figure 2:**
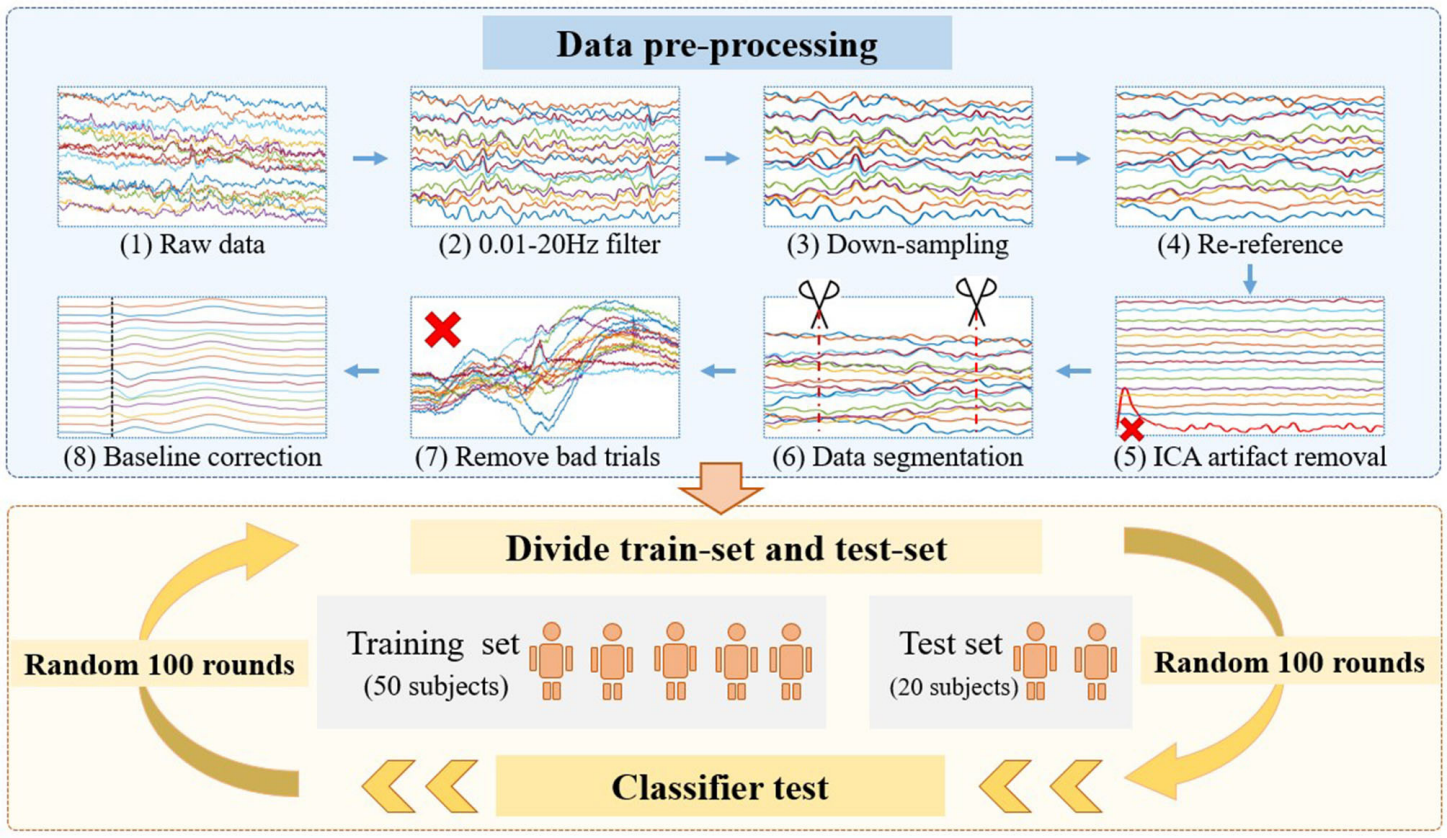
EEG identity authentication experimental flow chart.

Electroencephalogram identity authentication experimental flow chart is shown in Figure 2. In the preprocessing stage, 0.1–20 Hz band-pass filter was used to filter out slow drift and high-frequency noise offline. Band-pass filter consisted of low-pass and high-pass Chebyshev filters. The low-pass Chebyshev filters (order, 2; stopband starting frequency, 20 Hz; stopband cutoff frequency, 40 Hz; attenuation in the passband, 0.5 dB; attenuation in the stopband, 5 dB) were acquired through the built-in function of MATLAB. The high-pass Chebyshev filters (order, 2; stopband starting frequency, 0.01 Hz; stopband cutoff frequency, 0.5 Hz; attenuation in the passband, 0.5 dB; attenuation in the stopband, 10 dB) were also acquired. The original EEG data were down-sampled to 256 Hz to reduce the amount of data processing and calculation. Reference electrode standardization technique (REST) was applied for re-reference, and this method was considered to have higher accuracy in traceability and brain network analysis. The independent component analysis (ICA) was used to remove eye electrical artifacts in the signal. The ICA decomposition of EEG signals was realized by Fast-ICA toolbox of MATLAB, and the method applied the default parameters. We observed the decomposition waveform and determined the electrooculogram component, and this study only excluded the component of ocular artifacts. After the electrooculogram channel was set to zero, the rest of the ICA signal was converted back to the EEG signal. The test with amplitude >100 μv was automatically eliminated. EEG signals were cut into a period from −200 to 800 ms. The baseline of EEG signals was from −200 to 0 ms before stimulus presentation.

We invited 70 subjects (52 men and 18 women, age range of 18–25, standard deviation 1.7 years, 70 right-handed) to participate in the experiment, all of them were college students and had normal or corrected-to-normal visual ability. None of the subjects had a history of psychiatric illness or taking psychotropic drugs. The experimental design was approved by Ethics Committee of China National Digital Switching System Engineering and Technological Research Center. Each participant filled in the informed consent before the experiment and obtained a payment after completing the experiment. Compared with the current EEG database of 20–30 people, this experiment applied the relatively sufficient data of 70 people database. A total of 70 subjects were divided into training sets (50 subjects) and test sets (20 subjects). To avoid the contingency and increase the reproducibility, the participants in the training set and the test set were randomly selected. The result was the experiment average for 100 rounds of random training set and test set. To avoid the overfitting problem of identity authentication model, the two types of data sets have no overlap to test the robustness of the method.

The collected EEG signals were seriously disturbed by noise. The valuable ERP signal amplitude at 10^−6^ voltage, and any subtle influence seriously interferes with EEG data. Therefore, the applied EEG analysis and processing method needs to have good robustness. The ERP amplitude of valuable information in this experiment was below 10 μv, but the magnitude of other interference was above 100 μv. The collected EEG signals were mainly in the following three categories: environmental noise, electromyographic artifact, and psychological. Environmental noise includes equipment noise, power frequency interference, electromagnetic interference, etc. The artifacts include eye movements, head movements, and other EMG signals. In addition, EEG signals are affected by physical conditions such as mood, circadian clock, sleeping, and other psychological factors. To ensure the robustness of the proposed method, we have two special designs in our experiment. On the one hand, we collected a large-scale EEG database of 70 people, which tested the method's universality and effectiveness in the group. On the other hand, the experimental results were derived from 100 rounds randomly selected of training set and test set. The average results avoid the specificity and contingency of the proposed method.

### EEG 3D Presentation Form

**Figure 3:**
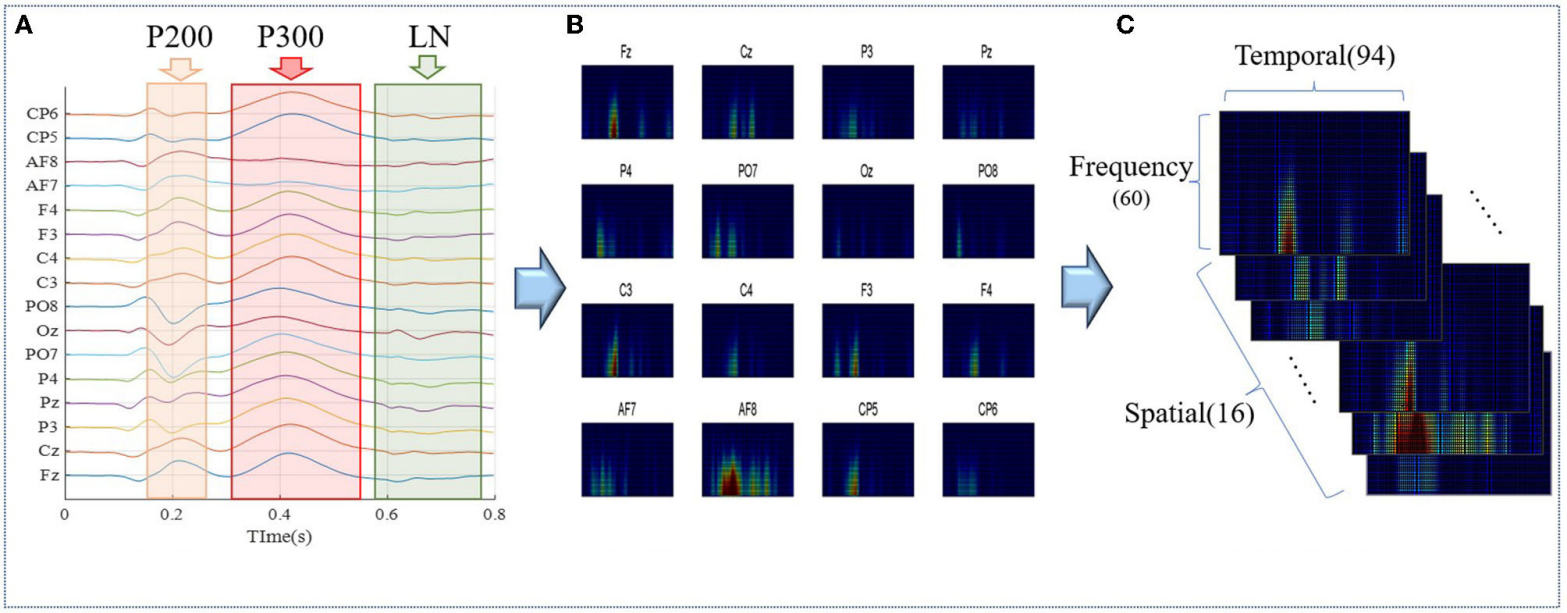
Three-dimensional transformation flow chart of EEG. **(A)** EEG after preprocessing. **(B)** Time-frequency energy diagram of separated channels. **(C)** Spatial–temporal–frequency domain combinations of EEG three-dimensional tensor.

To present more comprehensive and abundant EEG features, the 3D representation of EEG was assigned one dimension each for the spatial, temporal, and frequency domains. The three common feature domains were reflected in the three-dimensional representation, which fully characterizes individual identity from multiple perspectives. The neural activity recorded by EEG was shown in the time, frequency, and spatial domains. As shown in Figure 3A, the EEG contains P200, P300, and late negativity (LN) components, which represent the neuron activity of name stimuli. P200 shows the brain's pre-attention of visual stimuli, representing the early cognitive process. P300 is the most prominent component of EEG in this task. The presence of P300 means that the subjects' brains fully perceive name stimuli and stimulate large-scale, intense brain activity. Previous studies have found that LN belongs to post-processing of consciousness, indicating that stimulus materials are deeply processed in the brain. Figure 3B reveals the main energy centers at 375–625 ms in time domain and 0–10 Hz in frequency domain, which indicates that the brain's response to names is low frequency. In the spatial domain, the main energy was concentrated in the Fz, F3, F4, C3, and C4 channel. Brain activation is located in the frontal lobe and central area, which is responsible for advanced cognition and regulation of information.

The 3D transform process of EEG signals is shown in Figure 3. First, single-trial EEG data after preprocessing were separated according to different electrode channels. Second, the EEG signal time-frequency diagram conversion. The time-frequency diagram was obtained by the spectrum function in MATLAB. The spectrum parameters were set as follows: hamming window length 64, overlapping window length 57, and Fourier transform point 256. The size of the time-frequency graph of the processed single channel was 94 × 60, representing the time domain information of 0–800 ms and the frequency domain energy of 0–30 Hz, respectively. The stacking of 16 layers sensors formed the spatial representation. The spectrum parameter settings were constrained by the time-frequency characteristics of EEG signals and CNN input form. In terms of CNN input form, CNN was first outstanding in the field of image processing. Some studies have shown that CNN has outstanding effect on square images. Therefore, this experiment tried to generate time-frequency graphs close to square images. In terms of EEG characteristics, ERP signals showed high time information and low-frequency energy. Based on the above two points, we obtained the 94 × 60 square time-frequency diagram by setting the parameters (window length, 64; overlapping length, 57). In addition, the number of Fourier transform 256 points was the EEG sampling rate. Hamming window was widely used to reduce spectrum leakage and maintain good frequency resolution. Then, the spectrum of each channel is stacked to form a 16 × 94 × 60 three-dimensional input tensor, which represents the spatial, temporal, and frequency characteristics of EEG. Data processing decomposes EEG signals into mixed tensors of three feature domains, which is beneficial to feature fusion of neural network training.

### Multi-Scale Vision Convolution Module

**Figure 4:**
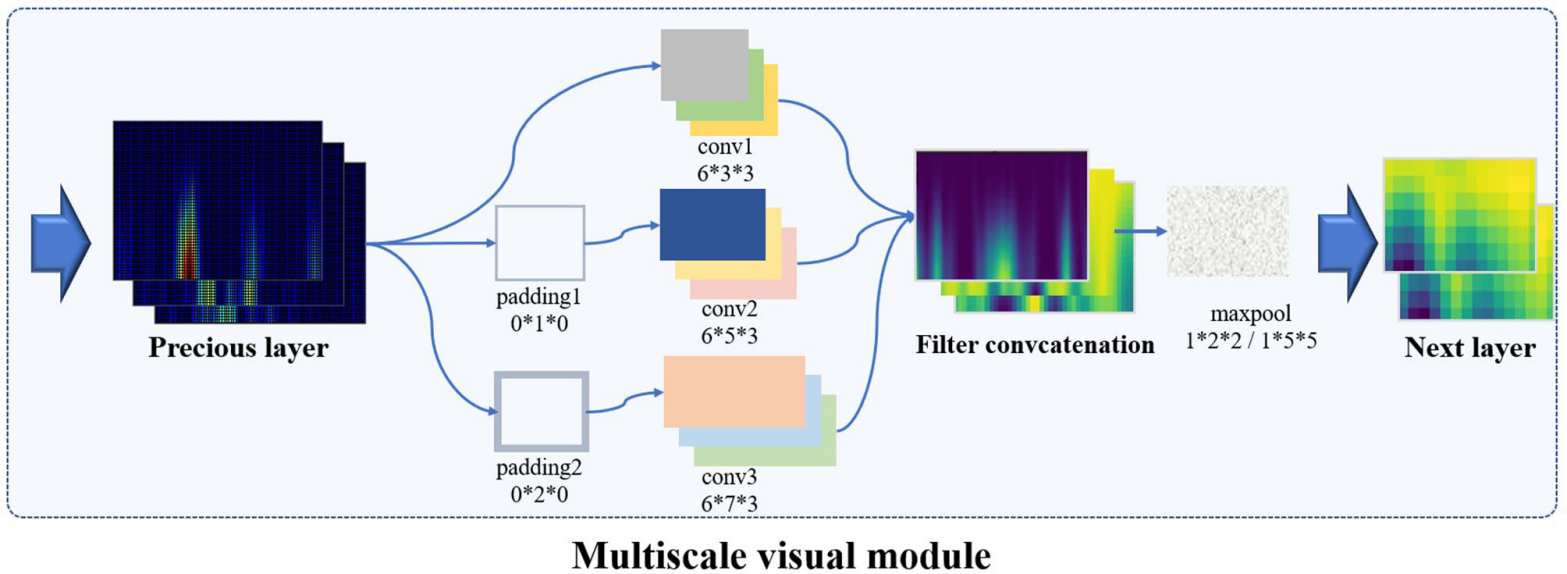
The architecture of multi-scale vision convolution module.

The individual identity feature of EEG was mainly extracted by multi-scale visual field convolution module, and multiple modules were spliced to form the overall network architecture. The previous data were applied to multiple branch operations, each branch connecting a convolution kernel of a unique scale. The multi-scale vision convolution module is shown in Figure 4. The multi-scale convolution processing flow included 1 × 1 convolution layer, branch structure, feature concatenation, and pooling.

The first input layer applied dimension reduction through 1 × 1 convolution layer, which reduced network parameters and integrates local correlation. Then, the 3D tensor was padded according to the scale of convolution kernel to meet the consistency of feature size after multi-scale convolution. To prevent gradient explosions or disappearances, the 3D convolution kernel was initialized by “kaiming_uniform.” Multi-branch convolution was an important structure for multi-scale feature extraction, and diversified convolution kernel combination strategy was the discussion focus in this study. Compared with the common square convolution kernel in the image field, considering the ERP signal characteristics of unbalanced information in temporal and frequency domains, a variety of rectangular convolution kernels with unequal edge lengths were explored in the study.

For the excellent multi-scale strategy [(6 × 3 × 3), (6 × 5 × 3), (6 × 7 × 3)], the experimental results are shown in Figure 4, and the 3D convolution kernels correspond to spatial × temporal × frequency domains, respectively. Multi-scale convolution kernel combination was conducive to capture EEG features from macro to detail, and multi-branch structure extracted diverse and complementary identity information. The feature output layer was regularized by batch normalization to improve the generalization ability and convergence speed of the model. The concatenate feature layer was reduced by maxpool, and the pooling kernel area decreased with the deepening of the network. The feature layer output contained comprehensive features extracted by multi-convolution kernel and transfers the three-dimensional information into the next stage.

### EEG Authentication Framework

**Figure 5:**
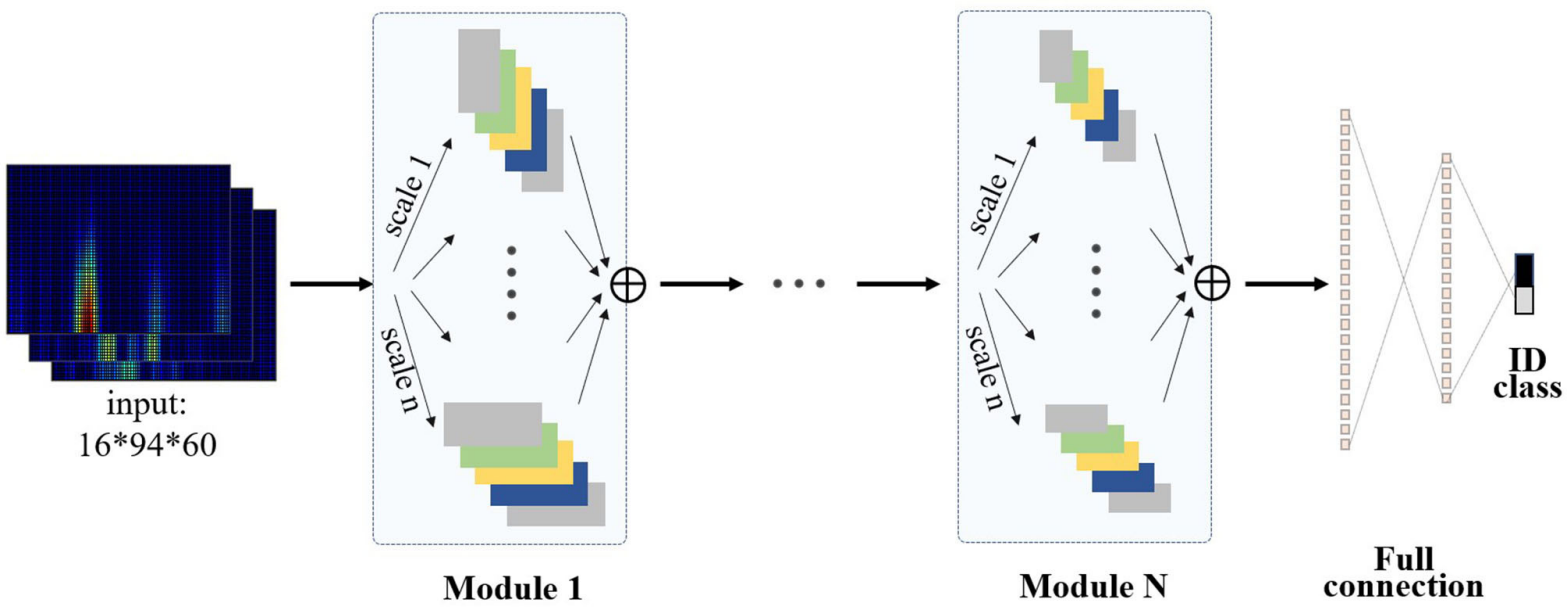
3D Multi-scale convolutional framework for EEG authentication.

The overall EEG authentication architecture was the stack and expansion of multi-scale convolution modules, and the number of branches and modules was flexibly combined. As shown in Figure 5, multi-branch structure within the module extracted diversity features, and multi-layer module stack can refine and combine the features. The multi-branch structure extracted diversity features in the basic module, and multi-layer module stacking can refine and combine the features. The end multi-scale convolution module flattens the features and then passes through a two-layer fully connected network of 768 × 32. The activation function used leaky rule (α =0 .01) to prevent dead neurons. To reduce the overfitting risk, dropout with a probability of 0.4 was inserted between the fully connected layers. The network parameters were set as follows: learning rate = 0.0005, epoch = 5, batch size = 50. The details of the network structure used in the experiment are shown in Table 1.

**Table 1:** Architecture of main neural networks for EEG authentication.

**Type**		**1D-simple**	**2D-simple**	**3D-simple**	**1D-Multi-**	**2D-Multi-**	**2D-Multi-**	**2D-Multi-**	**3D-Multi-**	**3D-Multi-**	**3D-Multi-**
				**scale-length**	**scale-width**	**scale-equal**	**scale-length**	**scale-width**	**scale-width**	**scale-equal**	**scale-length**
Multi-scale visual module(a)	Conv(a1)/ padding	(24) × 1 × 7/0 × 0	(24) × 9 × 7/0 × 0	(24) × 5 × 9 × 7/0 × 0	(24) × 1 × 7/0 × 2	(24) × 3 × 7/0 × 2	(24) × 7 × 7/2 × 2	(24) × 7 × 3/0 × 2 × 0	(24) × 6 × 3 × 7/0 × 0 × 2	(24) × 6 × 7 × 7/0 × 2 × 2	(24) × 6 × 7 × 3/0 × 2 × 0
	Conv(a2)/ padding	/	/	/	(24) × 1 × 5/0 × 1	(24) × 3 × 5/0 × 1	(24) × 5 × 5/1 × 1	(24) × 5 × 3/0 × 1 × 0	(24) × 6 × 3 × 5/0 × 0 × 1	(24) × 6 × 5 × 5/0 × 1 × 1	(24) × 6 × 5 × 3/0 × 1 × 0
	Conv(a3) /padding	/	/	/	(24) × 1 × 3/0 × 0	(24) × 3 × 3/0 × 0	(24) × 3 × 3/0 × 0	(24) × 3 × 3/0 × 0	(24) × 6 × 3 × 3/0 × 0 × 0	(24) × 6 × 3 × 3/0 × 0 × 0	(24) × 6 × 3 × 3/0 × 0 × 0
Max pool(a)	1 × 2	2 × 2	1 × 2 × 2	1 × 2	2 × 2	2 × 2	2 × 2	1 × 2 × 2	1 × 2 × 2	1 × 2 × 2
Multi-scale visual module(b)	Conv(b1) /padding	(72) × 1 × 7/0 × 0	(24) × 12 × 8/0 × 0	(72) × 7 × 12 × 8/0 × 0	(72) × 1 × 7/0 × 2	(72) × 3 × 7/0 × 2	(72) × 7 × 7/2 × 2	(72) × 7 × 3/0 × 2 × 0	(72) × 6 × 3 × 7/0 × 0 × 2	(72) × 6 × 7 × 7/0 × 2 × 2	(72) × 6 × 7 × 3/0 × 2 × 0
	Conv(b2) /padding	/	/	/	(72) × 1 × 5/0 × 1	(72) × 3 × 5/0 × 1	(72) × 5 × 5/1 × 1	(72) × 5 × 3/0 × 1 × 0	(72) × 6 × 3 × 5/0 × 0 × 1	(72) × 6 × 5 × 5/0 × 1 × 1	(72) × 6 × 5 × 3/0 × 1 × 0
	Conv(b3) /padding	/	/	/	(72) × 1 × 3/0 × 0	(72) × 3 × 3/0 × 0	(72) × 3 × 3/0 × 0	(72) × 3 × 3/0 × 0	(72) × 6 × 3 × 3/0 × 0 × 0	(72) × 6 × 3 × 3/0 × 0 × 0	(72) × 6 × 3 × 3/0 × 0 × 0
Max pool(b)	1 × 2	4 × 4	1 × 4 × 4	1 × 2	2 × 2	2 × 2	2 × 2	1 × 2 × 2	1 × 2 × 2	1 × 2 × 2
Multi-scale visual module(c)	Conv(c1) /padding	(72) × 1 × 7/0 × 0	(24) × 6 × 4/0 × 0	(72) × 6 × 6 × 4/0 × 0	(72) × 1 × 7/0 × 2	(72) × 3 × 7/0 × 2	(72) × 7 × 7/2 × 2	(72) × 7 × 3/2 × 0	(72) × 6 × 3 × 7/0 × 0 × 2	(72) × 6 × 7 × 7/0 × 2 × 2	(72) × 6 × 7 × 3/0 × 2 × 0
	Conv(c2) /padding	/	/	/	(72) × 1 × 5/0 × 1	(72) × 3 × 5/0 × 1	(72) × 5 × 5/1 × 1	(72) × 5 × 3/0 × 1 × 0	(72) × 6 × 3 × 5/0 × 0 × 1	(72) × 6 × 5 × 5/0 × 1 × 1	(72) × 6 × 5 × 3/0 × 1 × 0
	Conv(c3) /padding	/	/	/	(72) × 1 × 3/0 × 0	(72) × 3 × 3/0 × 0	(72) × 3 × 3/0 × 0	(72) × 3 × 3/0 × 0	(72) × 6 × 3 × 3/0 × 0 × 0	(72) × 6 × 3 × 3/0 × 0 × 0	(72) × 6 × 3 × 3/0 × 0 × 0
Max pool(c)	1 × 6	2 × 2	/	1 × 5	5 × 5	5 × 5	5 × 5	1 × 5 × 5	1 × 5 × 5	1 × 5 × 5
Dropout	0.4	0.4	0.4	0.4	0.4	0.4	0.4	0.4	0.4	0.4
Linear	16 × 3 × 72	72 × 3 × 2	72 × 3 × 2 × 1	16 × 4 × 72	4 × 2 × 72	4 × 2 × 72	4 × 2 × 72	1 × 4 × 2 × 72	1 × 4 × 2 × 72	1 × 4 × 2 × 72
Dropout	0.4	0.4	0.4	0.4	0.4	0.4	0.4	0.4	0.4	0.4
Linear	32	32	32	32	32	32	32	32	32	32
Softmax	2	2	2	2	2	2	2	2	2	2

It should be noted that the scale convolution framework should find a balance between network performance and training cost. Since the increase in the number of modules and branch structure will lead to a huge parameters' number, branch construction and module elimination are the main methods to reduce the computational and time consumption. The network framework in the study has flexibility and diversity. The experiment analyzes the several combination strategies of multi-branch and multi-layer modules and selects the architecture scheme that balances high performance and low time consumption.

## Results

### Single-Scale Kernel Performance

**Figure 6:**
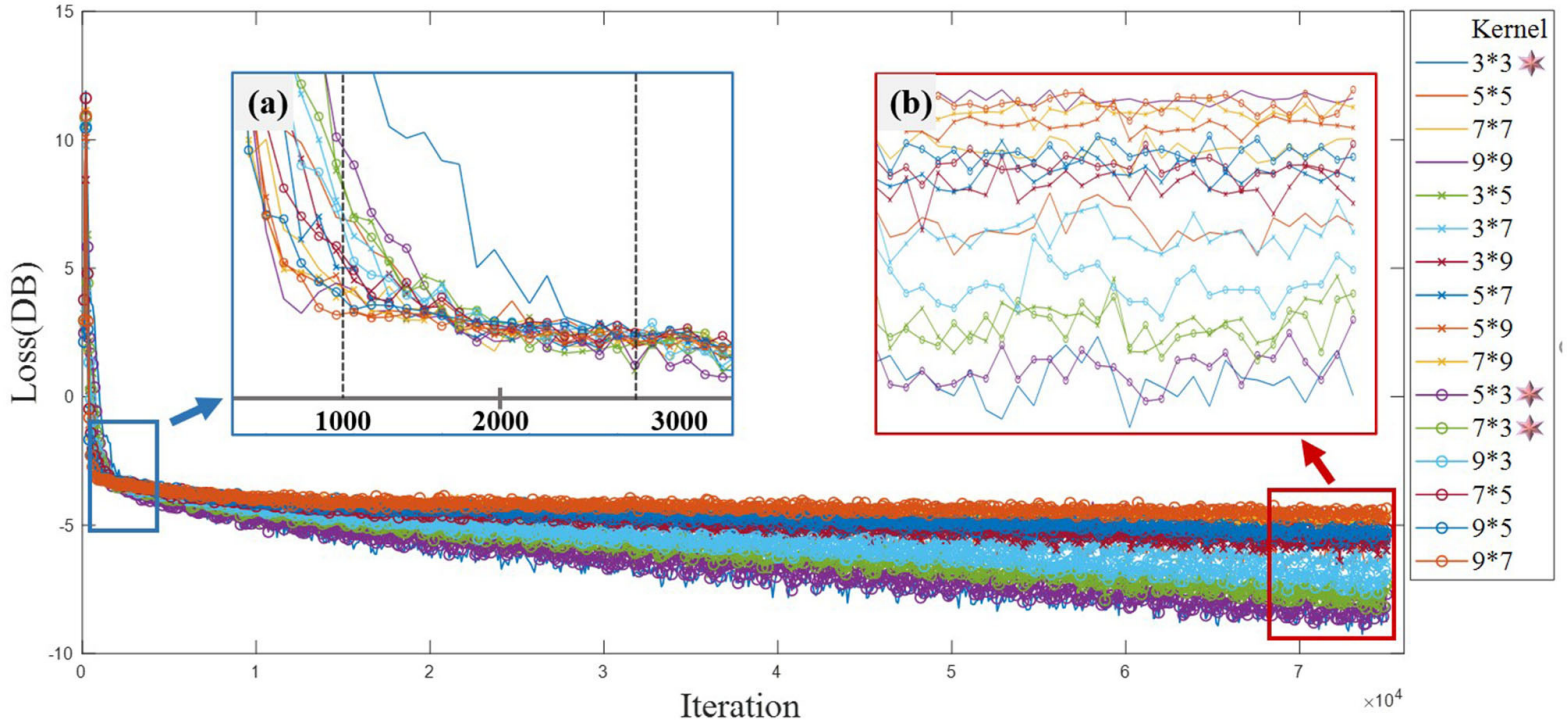
Network training loss of single-scale convolution kernel. **(a)** The important inflection points of network loss convergence. The loss functions of different convolution kernels decrease rapidly before 1,000 iterations. Additionally, the loss function curves of all convolution kernels tend to be flat before 3,000 rounds. **(b)** The loss function performance of different convolution kernels after multi-round iteration. Long-shape and small-size convolution kernels are suitable for feature extraction of ERP tasks.

**Figure 7:**
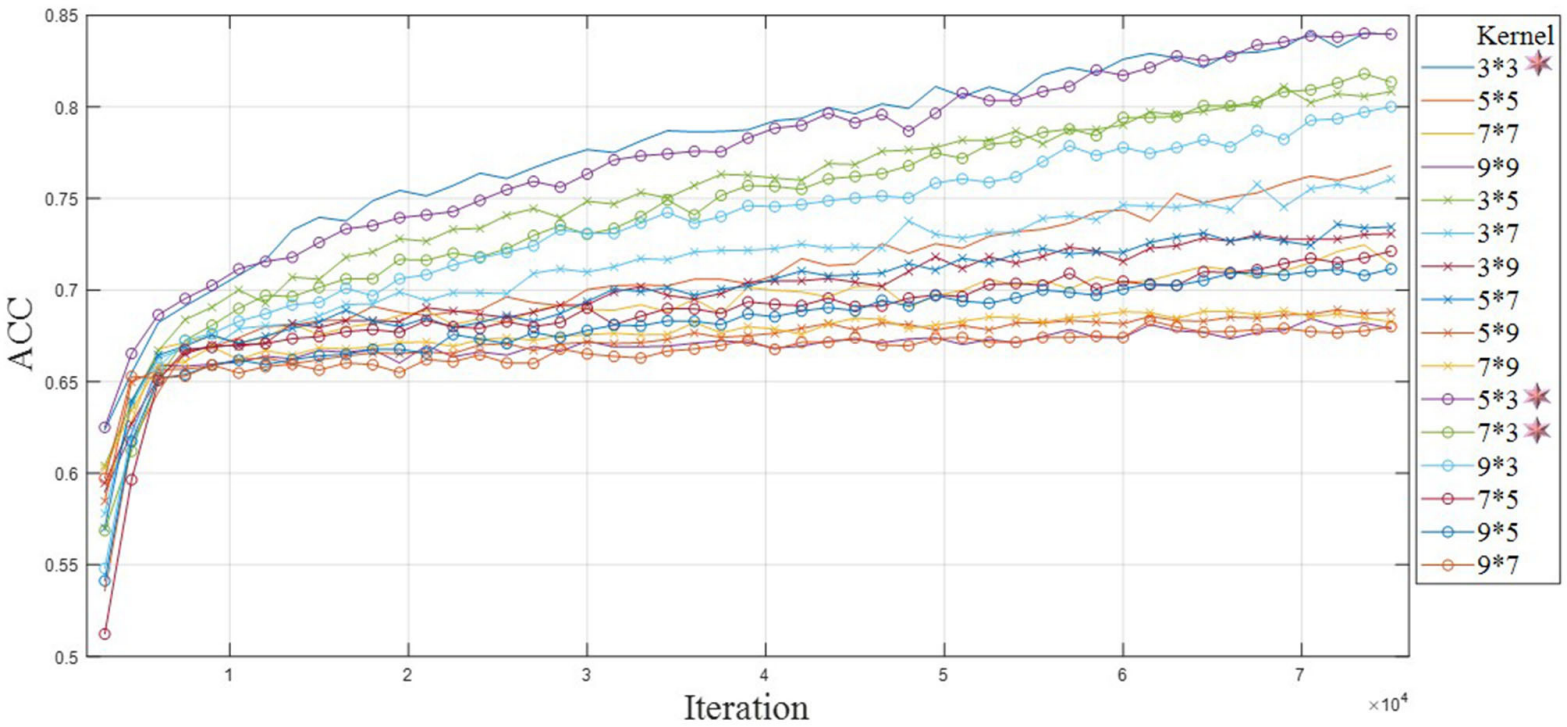
Test accuracy of single-scale convolution kernel.

The study explored the convolution kernel size suitable for EEG feature extraction and provided reference for multi-scale convolution kernel combination strategy. Figures 6, 7 show the training loss and classification performance of 16 single-scale convolution kernels. Due to the small spatial variability of 16 sensor space, the spatial depth of the fixed convolution kernel is 6. The study focuses on the feature information in time-frequency domain, and the convolution kernel size in the legend is time × frequency domain pixel).

Figure 6 shows the model training losses of convolution kernels with different scale. Figure 6A reveals important inflection points for convergence curves of different convolution kernels. The loss function curve shows two processes of rapid convergence and gentle decline. Model converges rapidly in the first 1,000 iterations. All convolution kernels reach the inflection point between 1,000 and 3,000 iterations, and the loss function decreases slowly after 3,000 rounds. As shown in Figure 6B, the top 3D kernels with the best convergence performance in time- frequency domain are 3 × 3, 5 × 3, and 7 × 3. The convolution kernels with the same area generally show that the long shape is better than the wide shape (e.g., 5 × 3 is better than 3 × 5), indicating that the attention degree of temporal characteristics mainly affects the convergence performance. The convolution kernels with different areas show that small size is better than large size (e.g., 3 × 3 is better than 7 × 7), indicating that the capture of EEG detail features by small size convolution kernel is beneficial for model training.

The classification test applied a 5-fold cross-validation method in the individual data set. A 5-fold cross-validation is a commonly used grouping test method in classification tasks, especially suitable for performance prediction of small-scale databases. The long duration of EEG experiments leads to subject discomfort and affects the data quality. The individual in single EEG experiment collected insufficient samples, and the samples' number of each type was <100. Individual model performance evaluation belongs to small-scale database testing. Therefore, we apply 5-fold cross-validation to reduce individual model overfitting. Figure 7 shows that the classification performance of different convolution kernels is consistent with the convergence performance, and the small rectangular convolution kernel has better classification performance in ERP feature extraction. The classification performance of small-size and long kernel shape is better.

Overall, the shape of convolution kernel affects the performance of the model more than the size, and the advantage order of convergence shapes under similar sizes is long, wide, and square shape. Rectangular shape is more suitable for ERP EEG feature model training, which is different from the experience of square convolution kernel in image processing field. Based on the above single-scale convolution kernel results, the smaller-scale convolution kernel is selected in the combined multi-scale convolution kernel strategy, and the shape difference in convolution kernel is retained for in-depth analysis. At present, our known literature retrieval has not found the research of the convolution kernel size in the EEG field. This study provides suggestions for convolution kernel selection of ERP tasks, including kernel size and strategy. Innovative research finds that rectangular cores and small sizes have better performance than common specifications in ERP tasks. In the construction of network framework, the convolution kernel can be selected and combined according to the design and strategy of this paper, which will be conducive to the improvement in EEG network performance.

### 3D-CNN Performance Evaluation

After quantitative analysis of the network performance of single-scale convolution kernel, the convolution kernel with excellent single-scale performance is composed of multi-scale convolution network. Taking the small size and non-square convolution kernel as the basic branch, seven kinds of convolution kernel combination strategies are proposed, and the convolution kernel combinations of long, wide, and square shapes are tested, respectively. One-dimensional multi-scale convolution model is vector input and only has long convolution kernel base. In addition, three commonly used single-scale CNN models and seven machine learning classification are compared in the experiment. The details of the network model are shown in Table 1.

**Figure 8:**
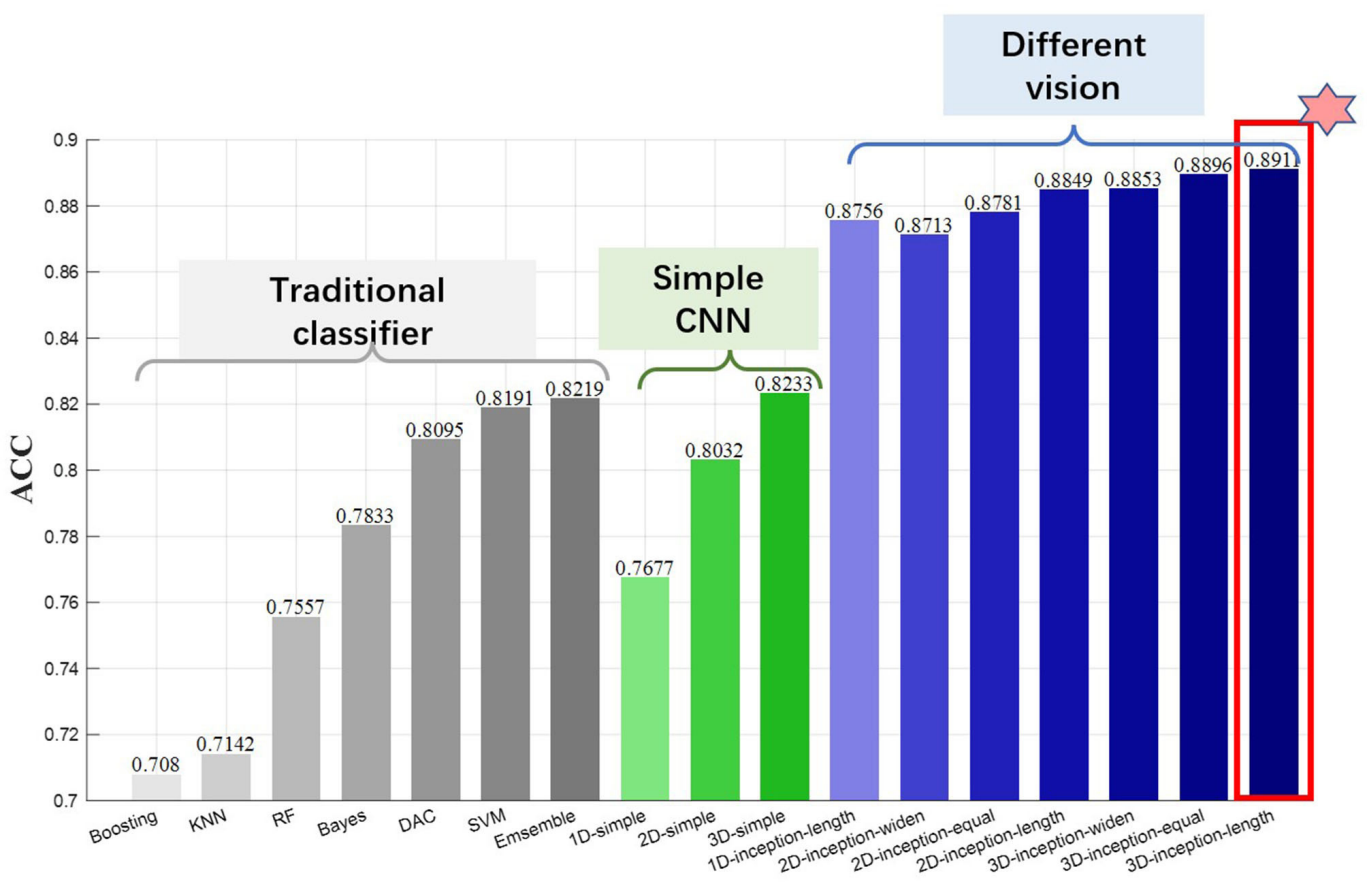
Classification performance of multiple authentication strategies.

The results in Figure 8 show that the classification performance of the traditional machine learning classifier is similar to that of the serial CNN model. The performance of traditional classifiers is quite different. Discriminant analysis classifier (DAC), support vector machine (SVM), and ensemble classifiers obtain higher classification accuracy, and their performance is close to the single-scale 3D-CNN network. The performance of the serial single-scale CNN model is affected by the data input dimension, and the fusion representation of multi-feature domains effectively improves the classification performance. The 3D single-scale convolutional network achieves 82.33% of the identity authentication accuracy, which is higher than the optimal traditional machine learning algorithm.

The convolution architecture of multi-scale visual field shows outstanding classification performance, and its multi-view feature extraction method obtains more than 5% of accuracy improvement compared with single-scale serial network. In addition, the multi-scale network also shows the advantages of high-dimensional feature fusion. The fusion features of multi-feature domain can extract individual identity information more accurately, and the accuracy rate increases with the increase of feature input dimension.

Compared with the 2D network, the 3D convolutional network proposed in the study has an improved accuracy rate, indicating that the spatial features extracted from the third dimension assist the classification decision. The cognitive process of self-name stimulation is divided into several stages: visual coding, semantic understanding, decision analysis, self-awareness, advanced cognition, neural feedback, etc. Different brain functional areas are responsible for the part of the cognitive process. Therefore, EEG signals of different electrodes have different contributions. However, the commonly used 2D-CNN lack the freedom degree of EEG spatial data, which makes it difficult to refine the importance of each electrode. The 3D-CNN is flexible in the third dimension, which helps to utilize spatial information of electrodes in different brain regions. In this article, the total number of channels is 16, and the convolution kernel size in the spatial dimension is 6. The 3D convolution kernel performs sliding window operation on the third dimension, and the convolution results are rich in 3D space layout. The 3D presentation helps the convolution network to obtain multi-dimensional optimization space, which is conducive to the optimization of network parameters and the improvement in classification performance. Since each functional area of the brain is responsible for different information processing tasks, the overall realization of the task requires the cooperation of multiple brain regions and the connection of the brain network, so the three-dimensional EEG presentation form is more suitable for the EEG biological characteristics. EEG acquisition equipment often uses multiple sensors to extract the neuron discharge signals of the whole brain, and the scalp electrical signal data between multiple sensors are cross-complementary, which effectively alleviates the spatial information lack of the single sensor. The 3D network model obtains the spatial correlation of EEG through the convolution vector of the third dimension and further extracts the spatial pattern of brain activity.

In the performance of the combination strategy of convolution kernel shape, the 2D and 3D networks show that long convolution kernel is better than wide and square. It is worth noting that the multi-scale convolution model with one-dimensional input achieves 87.56% of accuracy, and its classification effect is higher than that of the two-dimensional multi-scale network with wide shape. The advantages of rectangular convolution kernel in classification test are consistent with those of single convolution kernel, indicating that the rectangular kernel shape is more suitable for the ERP task network model.

Summarizing the results of the above three groups classification models, we find that the diverse features of multi-scale convolution kernel help to improve the classification performance, and the contribution of multi-scale architecture is better than that of multi-dimensional feature representation. In addition, a variety of long (temporal domain priority) convolution kernel combinations are suitable for EEG feature extraction.

### Analysis of Module Branch and Network Depth

**Figure 9:**
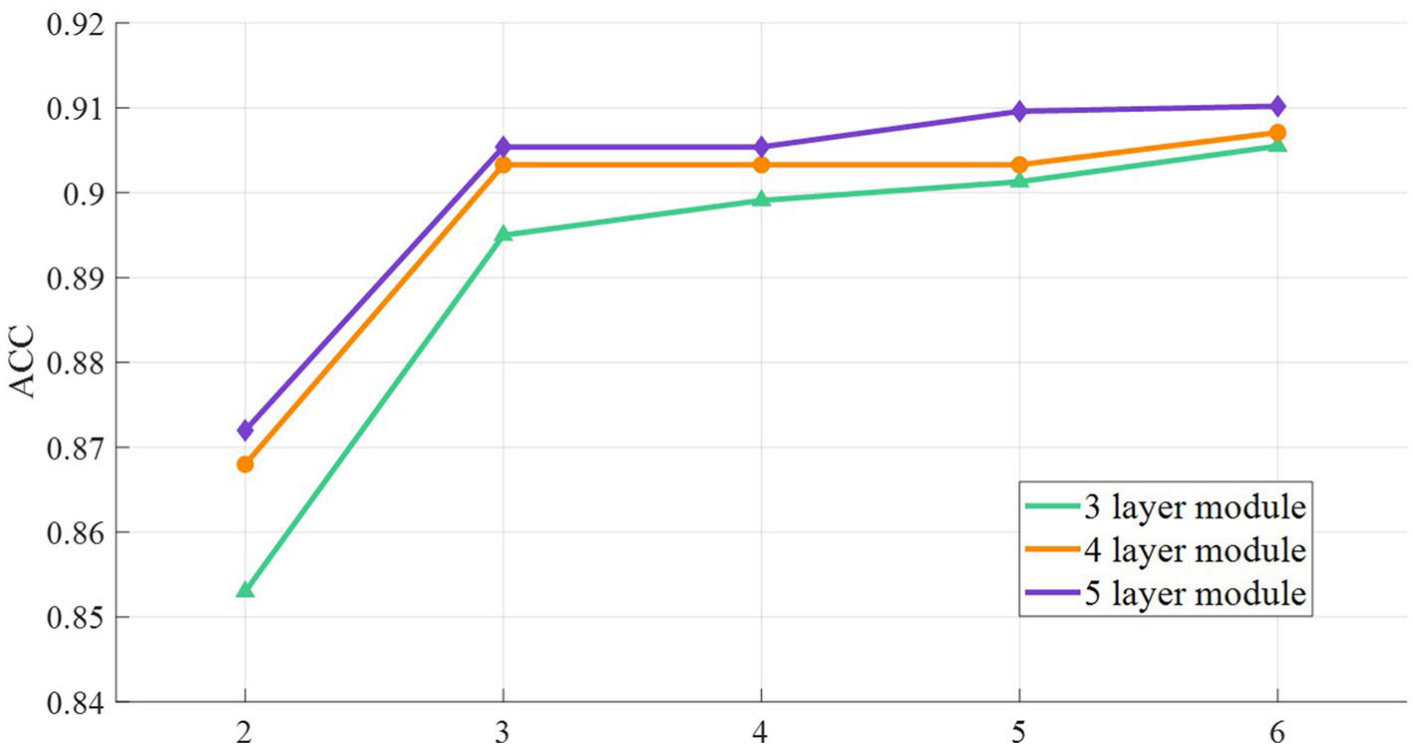
Classification performance of multi-layer, multi-branch network architecture.

To explore the influence of the breadth and depth of the network on the classification performance, the architecture of multi-branch and multi-layer networks was tested. Each branch assigns the operations of a single-scale convolution kernel. The multi-scale convolution kernel is expanded on the commonly used three branches, and the commonly used depth is selected for the network layer. The 2–6 branches are combined with the 3–5 module layers architecture, and the classification test results are shown in Figure 9.

The classification performance of multi-scale convolutional networks increases with the increase of layers and branches, indicating that the expansion of network parameters is conducive to feature extraction and classification. When 2 branches expand to 3 branches, the accuracy increases most significantly, and the increase in the branch number is limited after 3 branches. This shows that the convolution kernel of three branches can cover the basic EEG features, and too many branches increase the repeated acquisition and redundancy of features. In addition, the classification accuracy tends to be stable when the network depth is >3 layers, and the stacking module depth has limited performance improvement. In addition, complex networks require massive data and lead to overfitting.

The increase of branches and layers is accompanied by the improvement in training parameters, and the improvement in classification performance by complex networks is limited. The results show that the network architecture with three layers and three branches has good application cost performance, and the shallow network with fewer branches can obtain prominent identity authentication results.

### Feature Visualization of Multi-Scale Kernel

**Figure 10:**
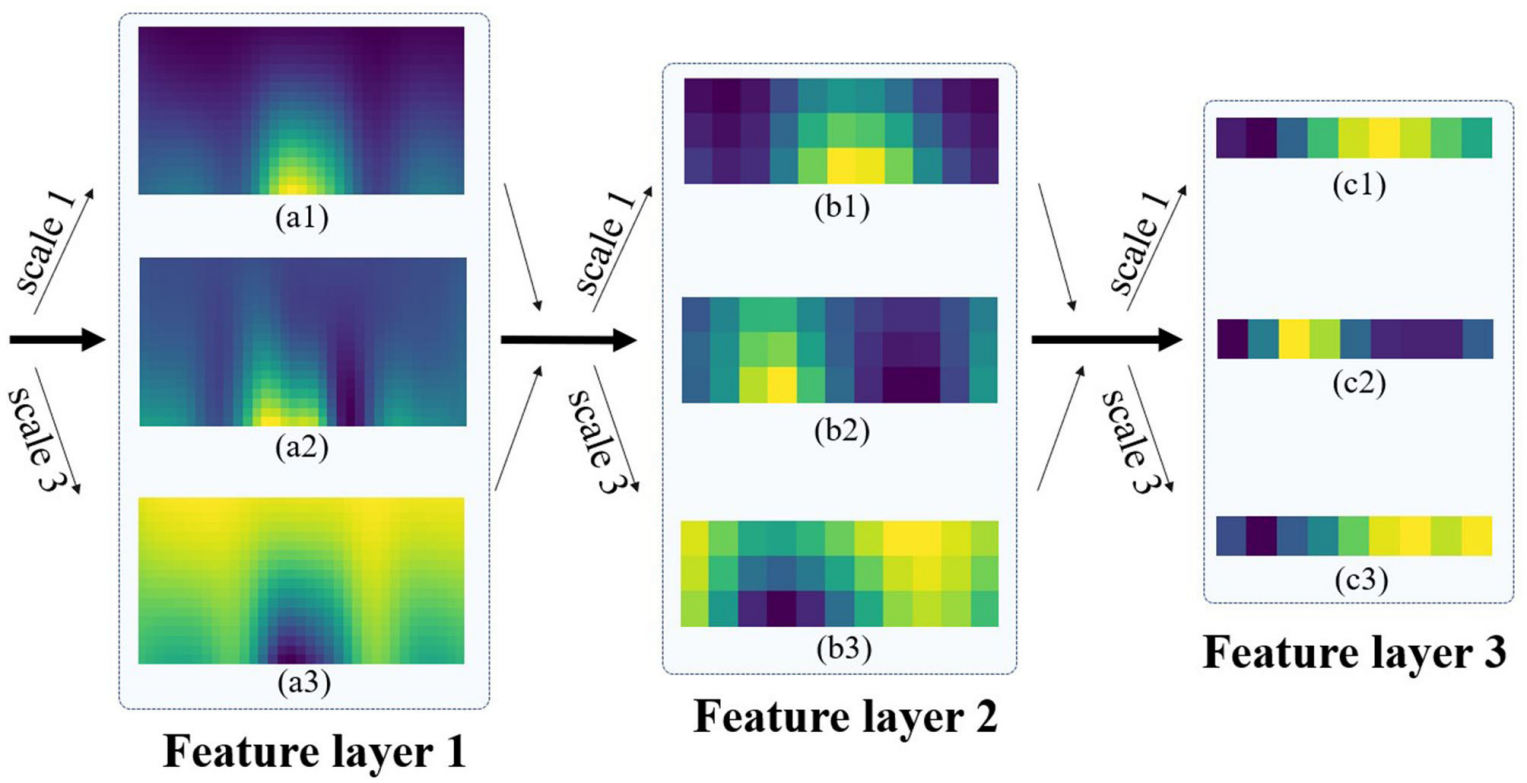
Visualization of intermediate feature layer of multi-scale convolution structure.

To analyze the extraction effect of multi-scale model for EEG identity features, we take three-branch and three model layers as an example to analyze the attention areas of different scale convolution kernels. Each branch outputs the feature heat map after convolution operation by the own scale and normalizes the attention intensity of the feature map by z-score. Figure 10 illustrates the feature layer extraction process of multi-scale network and shows the representative results of attention heat map.

For the results of the longitudinal single-layer feature, the multi-scale structure captures the diversity of EEG features, and the attention regions of different convolution kernels complement each other. We assume that the attention of multiple branches corresponds to the important components of EEG, such as the three convolution kernels of feature layer 1 capture the main features of ERP, respectively. Branch 1 corresponds to P300 component (a1), branch 2 corresponds to P200 component (a2), and branch 3 corresponds to early cognition and late negativity component (a3). In addition, EEG components affect convolution kernel size, the smaller convolution kernel (a1) size leads to the smaller span of captured features, and (a3) the longer convolution kernel size covers the large-scope features.

For the horizontal multi-layer feature inheritance, the bottom to the deep features represents good inheritance. The network effectively retains EEG features in layer-by-layer iteration and reduces the dimension of high-dimensional raw data to low-dimensional identity features. For example, (a1) (b1) (c1) feature maps retain the P300 component features in the conduction and convert the 2D time-frequency map of (a1) to one-dimensional vector of (c1). After compression in time domain and frequency domain, the P300 feature in (c1) still be clearly presented, and the corresponding position conforms to the temporal energy distribution. In addition, (c2) and (c3) also inherited the P200 and LN components of the bottom heat map, respectively. The results show that the multi-scale model integrates the early EEG features into the corresponding low-dimensional identity information.

The results in Figure 10 show that the features extracted by multi-scale model are diverse, complementary, and inheritable. The feature heat map is similar to the energy distribution of EEG in time domain, and the frequency domain features are merged and compressed, which is confirmed by the temporal priority of the early kernel result. In addition, we suggest that the branch number of the multi-scale module matches the main component number of ERP and select the multi kernel strategy of the corresponding ERP component size, which helps the branch structure of the multi-scale vision to focus on the EEG characteristics suitable for the respective scales.

### Time Consumption

**Figure 11:**
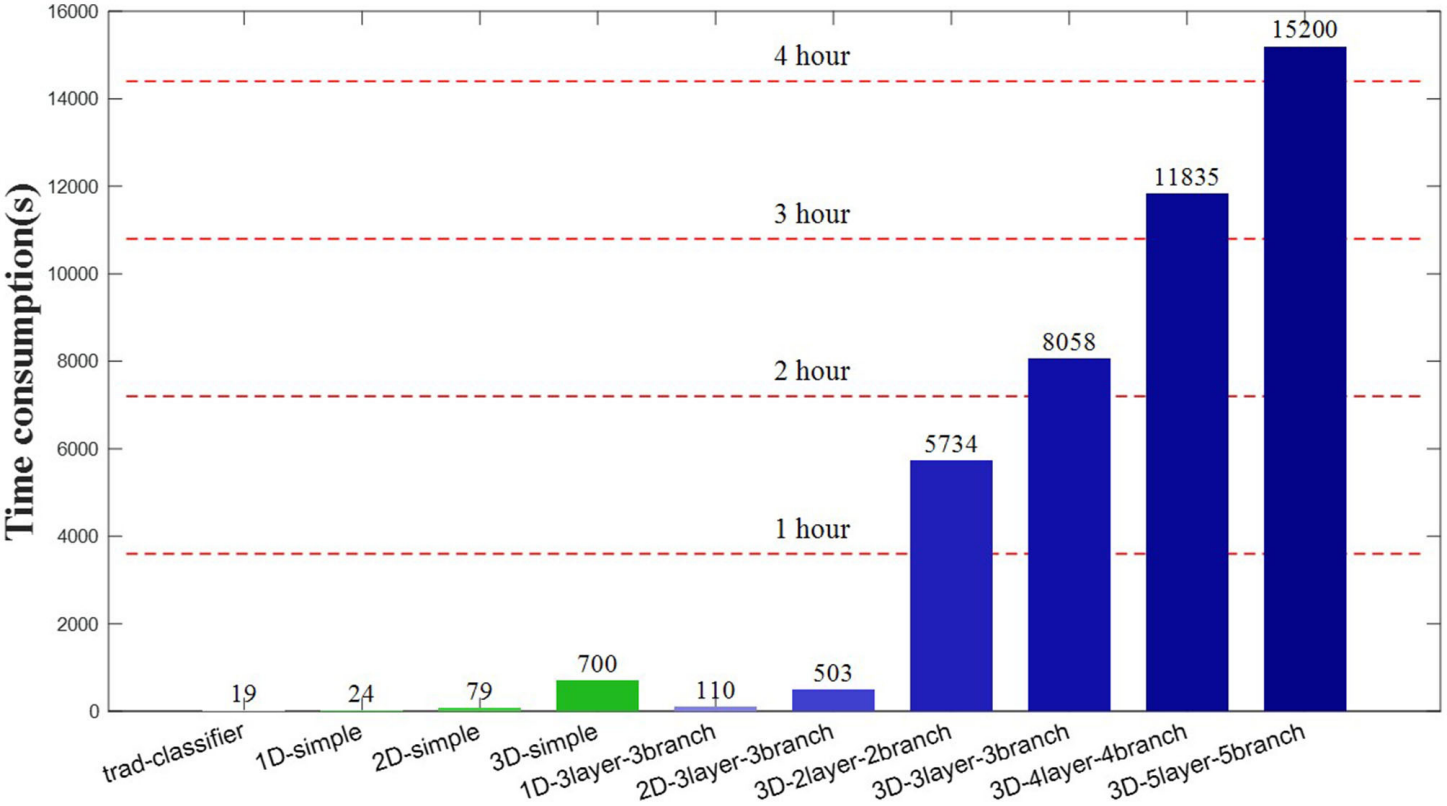
Training time consumption of different EEG authentication strategies.

The practical application of BCI system considers the computational power and time consumption of model construction, and the actual system needs to establish a balance between classification accuracy and time cost. Figure 11 shows the time-consuming training of the classification model for research and application, and the model training uses two high-performance CPU chips (Intel (R) Xeon (R) Gold 5118). The proposed model applies the large network parameters and the large batch size, and GPU is not applied due to insufficient memory.

The results show that the training time of the machine learning method is second level, and the serial single-scale convolution network is minute level, whereas the parallel multi-scale convolution network needs hour-level training consumption. The traditional machine learning method and serial single-scale convolution network have the advantages of low parameter and obtain effective classification performance in a short time, but the feature completeness and detection accuracy still have great room for improvement. The high-precision advantage of parallel multi-scale networks needs to sacrifice the computing consumption, which is lengthy and time-consuming for ordinary equipment. In addition, the complex model requires massive EEG samples; otherwise, it may lead to non-convergence or overfitting of model training.

There are some suggestions for reducing computational complexity and time-consuming for complex model frameworks. Data migration is a common method for training model optimization. A small amount of new data was inputted in the completed complex model, which obtains the low fine-tuning cost and the stability of the original model. In addition, the initialized parallel multi-scale network training should select a low-dimensional convolution framework. Figures 9, 11 show that the multi-scale network has similar classification accuracy, and the low-dimensional framework gains higher cost performance. Moreover, the increase in the branch number leads to the decrease in training speed. The results in Figure 9 show that the branch number should match the main component number of ERP, and the excess branch structure contributes less to the correct performance.

## Discussion

### Convolution Kernel Size Analysis

**Figure 12:**
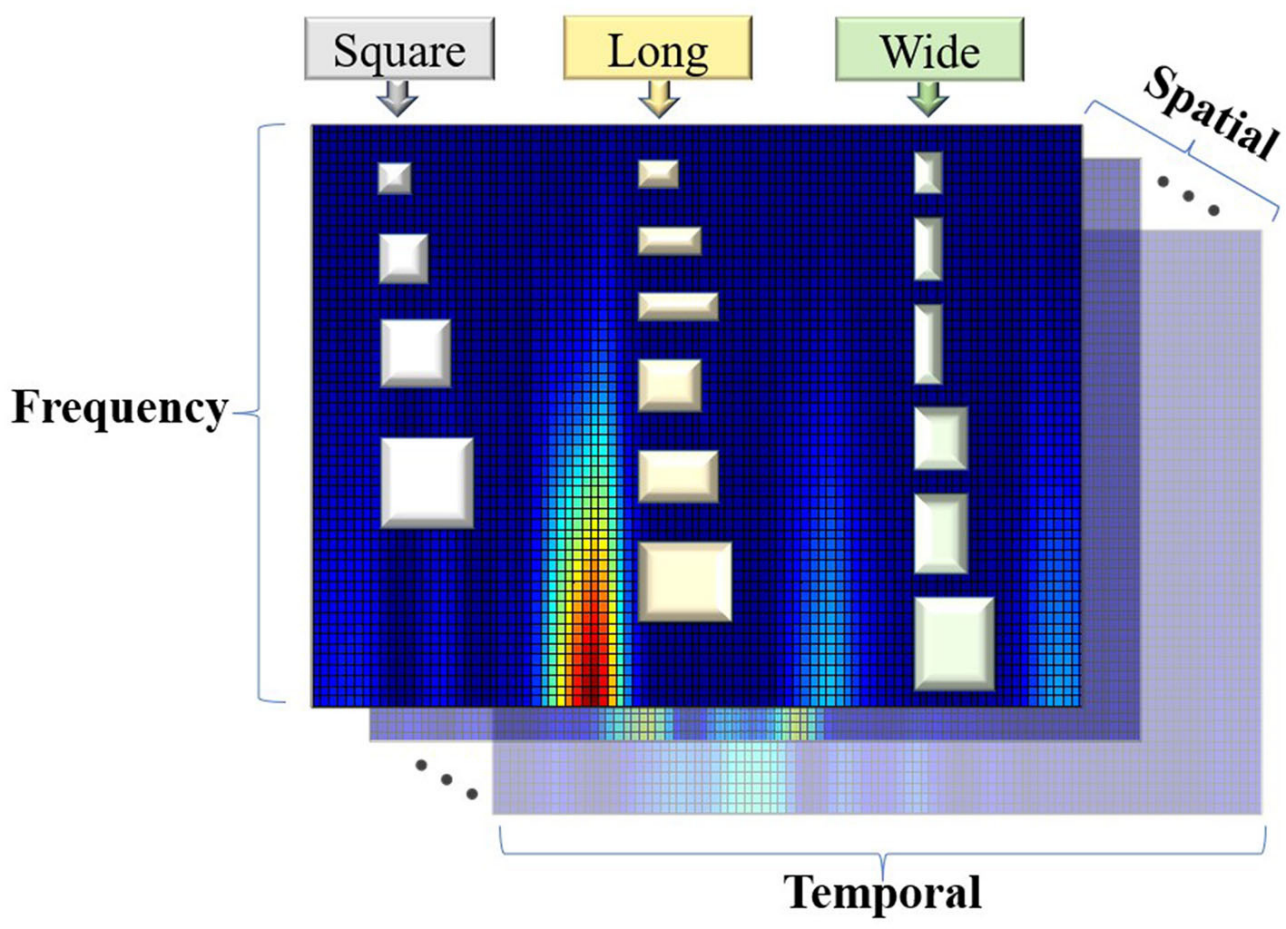
The convolution kernel corresponds to the real size of EEG data.

The results of single-scale and multi-scale model framework show that the size and shape of convolution kernel affect the network model performance. Figure 12 shows the kernel size corresponding to the actual EEG data. Table 2 quantizes the time-frequency area covered by the convolution kernel. The odd number of convolution kernel size is selected as the side length. The single pixel scale of EEG time-frequency diagram represents 8.5 ms × 0.5 Hz.

**Table 2:** Convolution kernel size corresponds to real EEG window size.

**Square kernel**	**Kernel size**	**3 ×3**	**5 ×5**	**7 ×7**	**9 ×9**	**/**	**/**
	**Actual size**	**25.5 ms ×1.5 Hz**	**42.5 ms ×2.5 Hz**	**59.5 ms ×3.5 Hz**	**76.5 ms ×5.5 Hz**	**/**	**/**
**Long kernel**	**Kernel size**	**5 ×3**	**7 ×3**	**9 ×3**	**7 ×5**	**9 ×5**	**9 ×7**
	**Actual size**	**42.5 ms ×1.5 Hz**	**59.5 ms ×1.5 Hz**	**76.5 ms ×1.5 Hz**	**59.5 ms ×2.5 Hz**	**76.5 ms ×2.5 Hz**	**76.5 ms ×3.5 Hz**
**Wide kernel**	**Kernel size**	**3 ×5**	**3 ×7**	**3 ×9**	**5 ×7**	**5 ×9**	**7 ×9**
	**Actual size**	**25.5 ms ×2.5 Hz**	**25.5 ms ×5.5 Hz**	**25.5 ms ×5.5 Hz**	**42.5 ms ×3.5 Hz**	**42.5 ms ×5.5 Hz**	**59.5 ms ×5.5 Hz**

*The bold value shows the best three convolution kernel shapes*.

The best three convolution dimensions of EEG authentication accuracy are 6 × 3 × 3, 6 × 5 × 3, and 6 × 7 × 3, which represents the convolution vector length of spatial, temporal, and frequency domains, respectively. The performance of long convolution kernel (temporal domain priority) is prominent, indicating that the network allocates a larger area of time domain feature. The rectangular convolution kernel shape has more advantages than the square convolution kernel in the image field. This is related to the ERP characteristic of diversity temporal components and low-frequency energy. A variety of convolution kernel sizes are tried in the experiment, and the results show that the larger convolution area in the time domain can obtain good convergence and accuracy. We speculate that there is more important information in time domain, which can help the network to extract effective EEG features. Large convolution kernel area can obtain larger receptive field, which helps to fully integrate the feature information in the field of vision. In addition, the larger convolution area in time domain represents more convolution parameters, which provides more optimization space for time domain feature extraction.

Most ERP studies tend to EEG feature extraction in temporal domain, while few studies on the low-frequency energy of the oddball paradigm. This is consistent with the temporal domain orientation of the convolution kernel size in this paper, and Figure 12 shows that ERP signal has the characteristics of high time-varying and low-frequency signals. This experiment is mainly aimed at the single-trial P300 EEG, and the performance of the long-during EEG convolution kernel still needs further research. It should be noted that the long kernel in this study corresponds to the high attention of time domain characteristics. If the input form is transformed, and the specific size should consider the actual corresponding time and frequency resolution. For the input time-frequency diagram transformation, changing the window length and overlap length of the time-frequency diagram parameters result in unit-valued size updates. The advantage of long convolution kernel mentioned in this work is 8.5 ms × 0.5 Hz unit size, and the unit size in the new experiment needs multiple transformation on this conclusion. For the input EEG task form transformation, EEG presents different features in time-locked and long-term tasks such as resting state and emotional detection. The rich frequency features of long-term tasks may lead to convolution kernel transform shape to capture frequency information.

Figure 6 shows that the smaller kernel size achieves better convergence. Table 2 shows that the convolution kernels with excessively large time span completely span the important components of the ERP, making it difficult to extract the changing details of the component dynamics. In addition, the energy of ERP signal is mainly concentrated in the low-frequency below 5 Hz, and the increase in frequency domain direction size reduces the capture of low-frequency feature details. A large number of studies on deep network structure also show that the performance of small-size convolution kernel is better than that of large-size convolution kernel. The vision of multi-layer small-size convolution kernel can replace the large-size convolution kernel and obtain the advantages of small parameters and conducive to training.

### Work Limitation

In EEG applications, the proposed network framework is mainly for time-locked tasks, especially P300 capture of target stimulus. However, long-during EEG tasks such as resting state, emotional recognition, and fatigue monitoring were not discussed. The proposed framework can be modified appropriately in future research to be applied to long-during EEG tasks. Due to the different EEG components caused by diverse stimuli, the convolution kernel size and shape of long-during EEG may be different from the time-locked task, which requires extensive attempts of multi-scale strategies for actual tasks. In addition, time-locked EEG tasks analyze the meaning of network feature layer by the P300 component localization, whereas the long-term stimulation usually lacks obvious observable components, which may lead to more difficulty in feature visualization and analysis.

In the future research of neural network systems, we will learn the advantages of excellent networks such as Vgg16, ResNet50, 101, and DarkNet53. Consistent with the strategy of stacking small convolution kernels in Vgg16 networks, this study also found excellent performance of small convolution kernels. Vgg16 focuses on the deep network of multilayers, and the proposed method applies the multi-branch breadth network. In the future network framework, the depth and breadth of the network structure can be combined to improve overall performance. ResNet applies the advantages of shortcut to alleviate the gradient disappearance problem and achieves outstanding performance in ultra-deep networks. The future network framework can apply the advantages of residual structure and insert shortcuts between multi-branch modules to achieve the deeper networks. It should be noted that insufficient EEG data may limit the performance of deep network, which should be considered in ultra-deep network training. The problem of insufficient EEG data can be solved by long-term collection or generation of artificial EEG samples. DarkNet53 is widely used in the image segmentation field, which applies the residual structure and convolution stride to replace the pooling layer. The proposed multi-scale 3D-CNN method can also simplify the network structure and reduce the time consumption by adjusting the convolution stride. The above methods inspire us with the network structure improvement in multiple perspectives. The proposed network should not only focus on multi-domain fusion and network breadth, but also improve the network depth and simplify the parameters.

## Conclusion

In this work, for the EEG identity authentication task, we proposed the feature domain fusion method of fusion temporal, frequency, and spatial domains. In addition, a corresponding 3D CNN framework with multi-scale model is constructed. The results show that sufficient feature fusion extracts individual EEG identity from multiple domains, which is conducive to the accurate identification of individual identity. In addition, the shape and size of convolution kernel are quantitatively analyzed according to the EEG signal characteristics. The research shows that the convergence and accuracy performance of 3 × 3 small-size kernel is outstanding in the time-frequency diagram, and the 3 × 5 and 3 × 7 rectangular-shape kernel with emphasis on time domain is more suitable for ERP tasks. In multi-scale architecture design, 3-layer 3-branch network matches the EEG components (P200, P300, LN) that balance the classification performance and time consumption. In summary, multi-scale convolution kernel parallelly extracts EEG identity features, which significantly improves 7% of accuracy in the identity authentication. The proposed novel network framework is suitable for EEG identity authentication, which can be transplanted and applied to various brain–computer interaction scenarios such as emotion recognition, fatigue driving, and neural rehabilitation.

## Data Availability Statement

The original contributions presented in the study are included in the article/supplementary material, further inquiries can be directed to the corresponding author/s.

## Author Contributions

RZ is mainly responsible for research design, data analysis, and manuscript writing of this study. YZ and KY are mainly responsible for research design. LT is mainly responsible for data collection and production of charts. JS is mainly responsible for contact subjects and participate in EEG data acquisition. RL is mainly responsible for experiment design and preparation. ZL is mainly responsible for experiment design and preparation, contact subjects, and participate in EEG data acquisition. BY is mainly responsible for data collection and manuscript modification. All authors contributed to the article and approved the submitted version.

## Funding

This work was supported in part by the National Key Research and Development Plan of China under Grant 2017YFB1002502, in part by the National Natural Science Foundation of China under Grant 61701089, and in part by the Natural Science Foundation of Henan Province of China under Grant 162300410333.

## Conflict of Interest

The authors declare that the research was conducted in the absence of any commercial or financial relationships that could be construed as a potential conflict of interest.

## Publisher's Note

All claims expressed in this article are solely those of the authors and do not necessarily represent those of their affiliated organizations, or those of the publisher, the editors and the reviewers. Any product that may be evaluated in this article, or claim that may be made by its manufacturer, is not guaranteed or endorsed by the publisher.
